# Interacting Quantum Atoms Method for Crystalline Solids

**DOI:** 10.1021/acs.jpca.1c06574

**Published:** 2021-10-01

**Authors:** Daniel Menéndez Crespo, Frank Richard Wagner, Evelio Francisco, Ángel Martín Pendás, Yuri Grin, Miroslav Kohout

**Affiliations:** † Max-Planck-Institut für Chemische Physik fester Stoffe, 01187 Dresden, Germany; ‡ Departamento de Química Física y Analítica, University of Oviedo, 33006 Oviedo, Spain

## Abstract

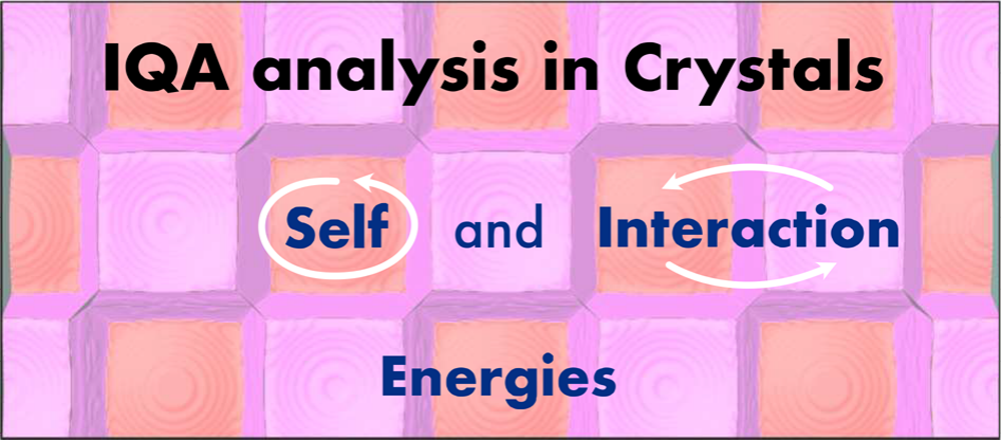

An implementation of the Interacting Quantum Atoms method for crystals is presented. It provides a real space energy decomposition of the energy of crystals in which all energy components are physically meaningful. The new package ChemInt enables one to compute intra-atomic and inter-atomic energies, as well as electron population measures used for quantitative description of chemical bonds in crystals. The implementation is tested and applied to characteristic molecular and crystalline systems with different types of bonding.

## Introduction

Nowadays, many interesting materials, e.g., intermetallic compounds, belong to the families of chemical systems, where the usual valence approaches based on pure ionicity, covalency with 2-center 2-electron bonds, or “metallic bonding” are no longer valid. The interplay of all these features challenges our understanding with the emergence of new bonding scenarios beyond the well-accepted concepts.^1−6^ Since chemical bonding analysis represents a way to understand inter-atomic interactions, it is also a way to understand material properties and their relations to specific electron counts or partial structures. For this sake, chemical bonding parameters like atomic charges and bond orders must be consistently related to their energetic counterparts.

This is achieved in the Interacting Quantum Atoms (IQA) method.^7−9^ It represents an ideal framework to investigate covalent and ionic interactions on an equal footing. The sum of the individual atomic interaction energies forms a part of the recoverable total energy of the system. All routinely used quantum mechanical packages yield the total electronic energy. Usually this energy is given in terms of kinetic and potential energy contributions. Additionally, the potential energy is split into Coulomb and exchange-correlation parts. For deeper insight into the mutual interactions between the atoms, a suitable analysis of the total energy, respectively the wave function and its components, must be performed. A number of tools and methods are available for this task, but only the periodic energy decomposition analysis (pEDA)^10^ has been used to decompose the total energy of extended systems. The decomposition is done in Hilbert space. Also the Crystal Orbital Hamilton Populations (COHP)^11^ analysis does the decomposition in Hilbert space but decomposes the band structure energy instead. An alternative and appealing method to decompose the total energy components into contributions of and between position-space atomic regions is the approach of IQA. Unique features of IQA are that it is orbital invariant, methodologically independent of the type of basis set, applicable to correlated wave functions, and does not depend on an external reference state.

The IQA scheme is based on the idea that the one- and two-electron energy integrals computed over the total coordinate space can be evaluated separately over any set of nonoverlapping spatial domains. The connection to a sound chemical description is achieved by the utilization of domains defined by the Quantum Theory of Atoms in Molecules (QTAIM), i.e., by the atomic basins determined by the density gradient field.^12^ With this, by successive integration of the one-electron components over each atomic basin, the total one-electron energy is decomposed into atomic contributions. The contributions describe the kinetic energy of the atomic domain as well as the attractive energy between the electrons and the nucleus enclosed within the atomic domain. The integration of the two-electron energy components over the atomic basins yields the intra-atomic and inter-atomic contributions that are used in the bonding analysis to judge the ionic and covalent bonding character. The whole procedure was adopted for molecular wave functions based on the Gauss-type of basis sets in the program Promolden.^13^

Until now, the IQA method has not been implemented for solids. Nevertheless, on the basis of careful calibration studies using a zeroth order approximation of the full IQA interaction energies, the so-called point-charge approximation of the covalent interaction energy and the ionic interaction energy (Madelung energy), was employed to understand chemical bonding and site occupation in ternary half-Heusler (MgAgAs-type) phases.^5^ Finally, this scheme was employed to predict new phases with this kind of structure.^6^ This may show the strength and scientific potential of the solid-state implementation of the exact methodology to be presented below.

The developed ChemInt software is coupled with the DGrid package.^14^ This extends the capability to evaluate the molecular Gauss-type orbitals also to the utilization of Slater-type based molecular wave functions, as used in the ADF package,^15^ as well as to access the numerical atomic orbitals expanding the wave functions for molecular and crystalline solid state systems computed with the FHI-aims code.^16^ ChemInt is also capable to compute the delocalization indices for all those types of basis sets, including crystalline systems.^17^ In particular, the bielectronic exchange energy integrals between position-space atomic regions for solid state wave functions are extremely challenging due to the huge number of participating orbitals, which made parallelization of ChemInt a necessary feature.

Theory and implementation of IQA for crystalline solids are outlined, reliability of the code is tested with an extensive set of molecules, and the method is applied to prototype solid-state compounds exhibiting different bonding scenarios.

## Methods

The total electronic energy of a molecule or solid with pairwise Coulomb interactions comprises the kinetic energy of the electrons *T*, electron–nucleus interactions *E*_ne_, electron–electron interactions *E*_ee_, and nucleus–nucleus interactions *E*_nn_
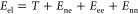
1Given access to the first order density matrix ρ_1_(***r***; ***r***′) and the pair density ρ_2_(***r***, ***r***′), the energy can be written as
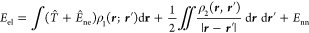
2Suppose that the space is partitioned into a set of domains that exhaustively recover the total volume and are mutually exclusive. Then, the total energy can be decomposed as
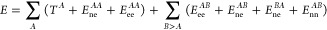
3The above energy terms can be written in compact form as the sum of domain self-energies and interdomain energies,
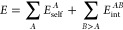
4Domain self-energies are
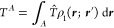
5
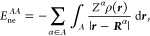
6where α ∈ *A* stands for all nuclei enclosed in domain *A*, and
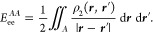
7Interdomain energies are
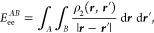
8
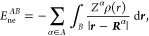
9and
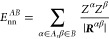
10Notice that intradomain electron–electron terms are halved to represent total energies whereas interdomain terms represent nonequivalent interaction energies.

The approach to the energy decomposition given by eq 4 considers the crystal to be formed by interacting atoms, molecules, etc. One may, otherwise, take a reference unit formed by one or more domains, embedded in the crystal. We collect, in an additive manner, *E* = *∑*_*G*_*E*(*G*), all energy contributions *E*(*G*) of reference unit *G*,
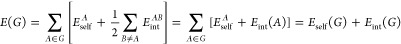
11The interaction energy for reference unit *G* shall be organized by coordination spheres *i*,
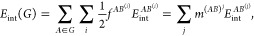
12where there are  number of *B* neighbors in the coordination sphere *i* of domain *A* of the reference unit. The last term condenses the sum over the coordination spheres for each domain in the reference unit into a single sum over coordination shells *j* for all domains in the reference unit and, with  interaction contributions  within that shell. The multiplicity factor  for the shell of those interactions (i.e., Li–Cl^(1)^ or Cl–Li^(1)^) takes the form
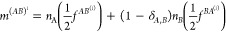
13where
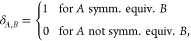
14and *n*_*A*_, *n*_*B*_ are the numbers of symmetry equivalent atoms *A* and *B* in the reference unit, respectively.

The pair density can be viewed as the product of two quasi-independent densities minus a term accounting for the difference with the true pair distribution ρ_2_(***r***, ***r***′) = ρ(***r***)ρ(***r***′) – ρ_*xc*_(***r***, ***r***′).^18^ This leads in eq 8 to the Coulomb energy contribution
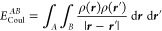
15and the exchange-correlation energy contribution
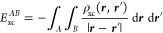
16With the exception of the exchange-correlation energy, all interdomain terms are classical. Thus, the interaction energy can be arranged as a sum of a classical electrostatic energy *E*_cl_^*AB*^ and a covalent energy *E*_xc_^*AB*^,
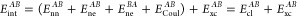
17

In the special case of the monodeterminantal wave function, the exchange-correlation density is directly given by the first order density matrix, ρ_xc_ = ρ_*x*_ = |ρ_1_(***r***; ***r***′)|^2^.

### Atomic Boundaries

Atomic domains defined by QTAIM theory are delimited by surfaces *S* that satisfy the zero-flux condition
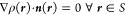
18where ρ(***r***) is the density at point ***r*** in the surface and ***n***(***r***) is a normal vector to the surface. The manifold of all steepest ascent paths
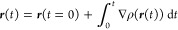
19terminating at given attractor (the common destination lim_*t*→*∞*_***r***(*t*)) constitutes an atomic (QTAIM) basin. In the following, the QTAIM basins are used as the spatial domains over which the energetical decomposition is performed.

We note at this point that a representation of the surface compatible with atomic-centered grids is required to use the multi/bipolar expansions presented in the following sections. To alleviate the expense of the computation, we precomputed a trust sphere using the algorithm described by Rodríguez et al.^19^

Of fundamental relevance for solid state systems is achieving electroneutrality in the unit cell. In contrast to molecules, all atomic basins determined for a solid state system have finite volume. Thus, the atomic basins must recover the unit cell volume, a circumstance that seems to be problematic in case of atom-centered radial grids. We decided to use radial rays with symmetric spherical *t*-designs.^20^

### Energy Integrals in the IQA Framework

The energy integrals for a system with perfect periodicity are performed over complex crystal orbitals ϕ_*n*,***k***_ for given band index *n* and reciprocal vector ***k***
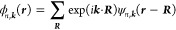
with ***R*** given by the unit cell vectors and the linear combination ψ_*n*,***k***_(***r*** – ***R***) = *∑*_*j*_*c*_*n*,*j*_(***k***) χ_*j*_(***r*** – ***a***_*j*_) of atomic orbitals χ_*j*_(***r*** – ***a***_*j*_), centered at ***a***_*j*_ = ***r***_*j*_ + ***R***, and with coeficients *c*_*n*,*j*_(***k***) to be determined. In this paper, electronic structure calculations were done with FHI-aims, utilizing numerical atomic orbitals.

The band and translational symmetry labels are condensed here to a single label *i* ≡ (*n*, ***k***). With this, the crystal orbitals ϕ_*i*_ determine the electron density  as well as exchange pair density , where  are overlap densities.

The density is replaced in eq 15, and the exchange pair density in eq 16,
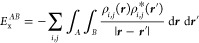
20with domains being now QTAIM atoms. Recall from eq 7 that *B* = *A* integrals have a  prefactor.

Intra-atomic Coulomb integrals (*A* = *B*) are computed with the Laplace expansion of 1/|***r*** – ***r***′| around the position of the atomic nucleus^7^
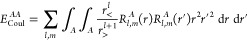
21where  is the angular (*r̂* = (θ,ϕ)) integral of the atomic density
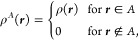
22weighted by real spherical harmonic *S*_*l*,*m*_. The discriminant , with *r*_<_ =  min(*r*, *r*′) and *r*_>_ =  max(*r*, *r*′), ensures the convergence of the expansion.

The expansion is valid also for the exchange integral^7^
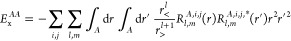
23where . Atomic overlap densities ρ_*i*,*j*_^*A*^ are zero outside of the basin like the atomic density above.

The integral evaluation is more efficiently performed in the basis of overlap densities . The exchange term is diagonal in this basis with coefficients *d*_*ij*_ = 2(2 – δ_*ij*_) for monodeterminantal wave functions,^9^ and no monadic diagonalization is required.

Two-center integrals (*A* ≠ *B*) are expanded around two poles, one at each atomic position, with a bipolar expansion^21−23^
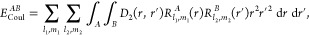
24where  replaces  as the discriminant.^7^

The exchange integral between two different basins is
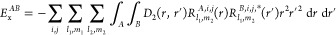
25

Once the electronic structure problem is solved by whatever method, the self-energy of each atom (or fragment) in the unit cell is obtained. The interaction energy of each of them, e.g., *A*, with the rest of atoms of the lattice, appropriately classified by increasing distance, *B*, is obtained (). This infinite sum contains quickly convergent terms *E*_x_^*AB*^, which decay exponentially in insulators and polynomially in metals, and a potentially divergent series made up of *E*_cl_^*AB*^ electrostatic interactions. By separating *E*_cl_ into a short-range, penetration-like exponentially decaying term and a multipolar one that is summed via the Ewald construction, one gets rid of divergences. We have applied the Ewald construction with the QTAIM monopoles (net atomic charges) via the Environ program^24^ and have summed directly the higher order multipolar corrections from the IQA integrations up to near neighbors. Even if the QTAIM monopoles are zero, those terms remain and constitute the well-known electrostatic interaction between uncharged atoms or molecules.

### Approximations Based on Population Measures

Inter-atomic bielectronic integrals are a particularly expensive step. As seen in eqs 24 and 25, they involve a bipolar expansion. For classic electrostatic energy integrals of distant basins it is equivalent to use a multipolar expansion^25^
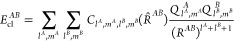
26where
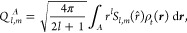
27is a real multipole moment of the net charge density ρ_*t*_(***r***) = *∑*_*A*_*Z*^*A*^δ(***r*** – ***R***^*A*^) – ρ(***r***) in basin *A*.  is an angular factor (cf. eq 25 in ref (7)). Technically, this approximation is invalid if the circumsphere of the basin *A* (centered at the nuclear position) intersects the circumsphere of basin *B* (with center at nucleus *B*). As a remark, solid state systems normally have compact basins. This limits the extent of the spheres, and it is safe to say that classic interactions with neighbor atoms of the second and higher coordination spheres are well approximated with a multipolar expansion in these cases. Also, internuclear distances in the solid state systems are often large, exceeding 2 Å.

On the other hand, exchange integrals were proven numerically to lead to asymptotic convergent multipolar expansions, with low-order terms being a good approximation for second and further nearest neighbors.^26^ The appropriate expression has the same structure as for classic interactions
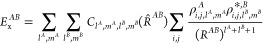
28but in terms of the sum of overlap density multipoles
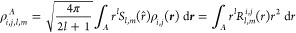
29

Further approximation of bielectronic integrals is to consider only the first term in the multipolar expansion. For the classic electrostatic term it is the same as assuming spherical density distribution in the atomic domain. By Gauss law, a point charge located at the nucleus position (for A and B) produces a physically equivalent classic force. The classic electrostatic energy,
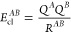
30is proportional to the product of QTAIM net charges  for both basins, where  is the average electron population in basin *A*.

Inter-atomic exchange integrals also admit a similar expression^26^
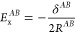
31that instead involves the delocalization index (DI) δ^*AB*^ between the atomic domains A and B
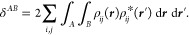
32The delocalization index measures the number of electrons shared between domains (*A* and *B*) and is complemented by the localization index (LI)
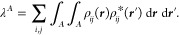
33Localization and delocalization indices must obey the following sum rule
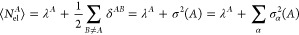
34The fluctuation of electron population σ^2^(*A*) in basin *A* is the result of electron pair sharing with atoms of the first coordination shell σ_1_^2^(*A*), second coordination shell σ_2_^2^(*A*), and so on.

Expressions 30 and 31 are the basis for our more generic scaled point-charge approximation (sPCA) for classic ionic and exchange (covalent) inter-atomic interactions. The scaling parameters *s*_cl_ and *s*_*x*_ approximate the exact classic and exchange energy, respectively, with a scaled zeroth order multipolar approximation:
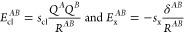
35

## Computational Details

Density functional theory calculations (PBE functional^27^) for crystalline solids were performed with FHI-aims, an all-electron full-potential electronic-structure code. The numeric atom-centered basis sets used were of standard “tight” type. The computed unit cells and Brillouin zone samplings are listed in Table 1. An external output option of FHI-aims (version 200112) is used to generate DGrid compatible wave function information. ChemInt interfaces DGrid to evaluate wave function properties at discrete points in space, determines the atomic QTAIM basins, and performs the IQA analysis with this space partitioning.

**Table 1:** Crystallographic Information and Brillouin Zone Sampling for the Calculated Materials^a^

system	space group type	lattice parameters [Å]	calculated cell	*k*-point mesh
β-N_2_^b^	*P*6_3_/*mmc*	*a* = 4.050^c^	1 × 1 × 1	5 × 5 × 3
		*c* = 6.604		
CO_2_	*Pa*3̅	*a* = 5.4942^35^	1 × 1 × 1	4 × 4 × 4
diamond	*Fd*3̅*m*	*a* = 3.566606^36^	1 × 1 × 1	4 × 4 × 4
BN (zincblende)	*F*4̅3*m*	*a* = 3.61^37^	1 × 1 × 1	4 × 4 × 4
graphite	*P*6_3_/*mmc*	*a* = 2.464^38^	2 × 2 × 1	4 × 4 × 3
		*c* = 6.711		
BN-b	*P*6_3_/*mmc*	*a* = 2.504323^39^	2 × 2 × 1	4 × 4 × 3
		*c* = 6.658852		
MgB_2_	*P*6/*mmm*	*a* = 3.0846^40^	2 × 2 × 1	3 × 3 × 5
		*c* = 3.5199		
LiCl	*Fm*3̅*m*	*a* = 5.12952^41^	1 × 1 × 1	4 × 4 × 4
NaCl	*Fm*3̅*m*	*a* = 5.7915^42^	1 × 1 × 1	4 × 4 × 4
MgO	*Fm*3̅*m*	*a* = 4.213^43^	1 × 1 × 1	4 × 4 × 4
Al	*Fm*3̅*m*	*a* = 4.0494^38^	1 × 1 × 1	4 × 4 × 4
Na	*Im*3*®m*	*a* = 4.235^44^	1 × 1 × 1	4 × 4 × 4

a Lattice parameters are given for the crystallographic cell, and calculated cells are specified as multiples of the crystallographic cell.

b The *z* coordinate for the Wyckoff site is calculated assuming a bond length of 1.108 Å and that the molecule center is located at the symmetry center of the space group *P*6_3_/*mmc*, as in ref (45).

c Lattice parameters taken from ref (46).

The set of molecules taken for the validation were computed with GAMESS,^28^ ADF,^15^ and FHI-aims,^16^ employing HF, LDA,^29−31^ PBE,^27^ BLYP,^32,33^ and B3LYP^34^ functionals. The basis set taken within each program is cc-pvtz (GAMESS), TZ2P (ADF), and standard “tight” (FHI-aims). For each molecule, the geometry was optimized before integrating the energy components. The geometry was fixed for molecules used for comparison against the solid phase (N_2_, CO_2_, neopentane, and phenalene). Only the four central atoms of phenalene were fixed while the positions of the other atoms were optimized.

## Results and Discussion

IQA energy terms are examined for a number of molecular systems to evaluate the energy error. In subsequent exemplary application studies, some prototype compounds (diamond and zincblende structures, honeycomb networks, rocksalt structures, closest packings) covering covalent, ionic, and metallic situations were investigated.

### Validation of the Implementation

Three approaches are explored to validate our implementation. First, similar results should be obtained with alternative implementations when comparable (molecular wave functions expanded with GTOs). In this document only the ethane system is shown (Table 2), but the equivalence was tested also with other simple systems reaching always similar accuracy. Second, properties like the volume, exchange-correlation energy, and total energy in the cell were checked against FHI-aims. The total energy requires an exhaustive computation of long- range interactions to be recovered. The electron count sum for the (crystallographic) unit cell provide a third way to check the validity of our implementation. Only the first is examined in this section and the other two in the next sections.

**Table 2:** Total Energy Components of Ethane Computed with ChemInt, Promolden, and GAMESS^a^

energy	ChemInt	Promolden	GAMESS
total energy, *E*	–79.731	–79.732	–79.730
kinetic energy, *T*	79.243	79.243	–79.244
total potential energy, *E*_ne_	–158.974	–158.975	–158.974
electron–electron energy, *E*_ee_	67.551	67.552	67.551
Coulomb energy, *E*_Coul_	80.528	80.529	80.528
exchange-correlation energy, *E*_xc_	–12.977	–12.977	–12.977

a The evaluated number of electrons per formula unit are 17.9992 (ChemInt) and 18.0001 (Promolden). *E*_*x*_ was re-scaled from the integration of the PBE functional.^47^ Energy in Ha units.

For the first check, we take the same molecular wave function and perform an energy decomposition analysis with two independent IQA implementations: our code and the Promolden code.^13^ We see that the integrated quantities have similar accuracy to those of Promolden and are consistent, beyond the chemical accuracy of approximately 1 mHa, with accurate full space integrations given by GAMESS (Table 2).

**Figure 1:**
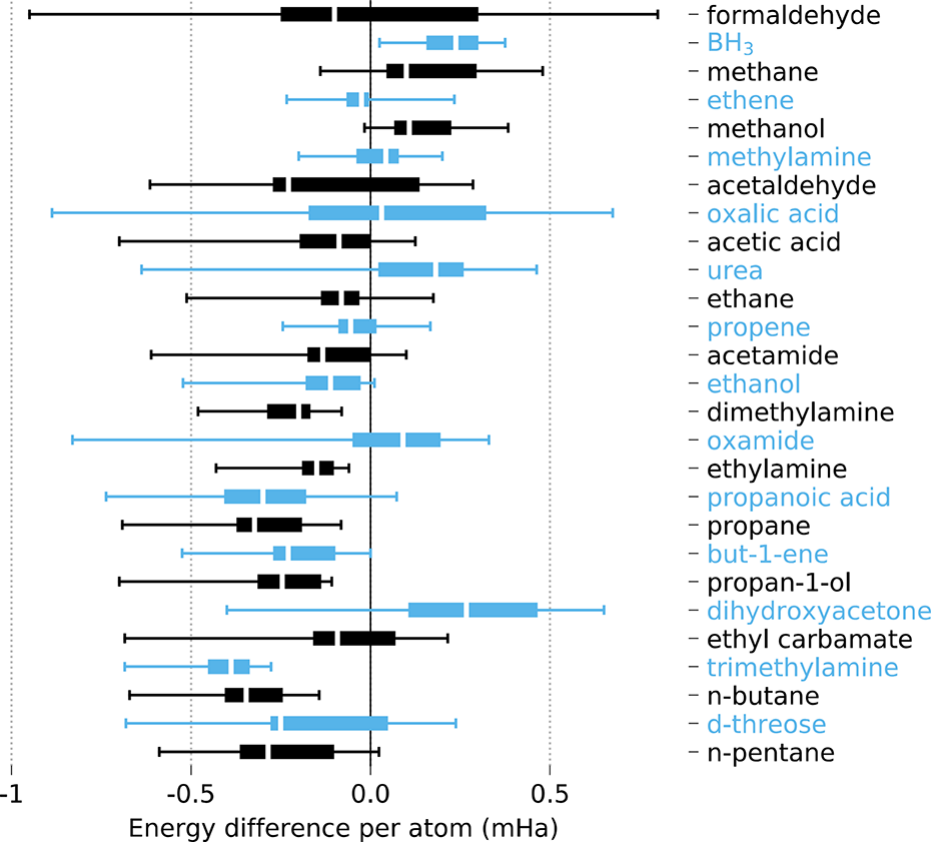
Energy difference (per atom) between the reconstructed IQA energy and the SCF energy (energy error). The white band denotes the median. Boxes delimit the lower and upper quartiles Q3 and Q4, and fences delimit all energy errors from a sample that takes wave functions computed with GAMESS, ADF, or FHI-aims, employing HF, LDA, PBE, BLYP, and B3LYP functionals for every system. *E*_*x*_ was rescaled from the integration of the corresponding functional.^47^ All errors fall below 1 mHa.

The precision of IQA decomposition is mainly determined by errors in the atomic boundary, the quality of the grid, and the truncation of the Laplace/bipolar expansion. Another possible source of error can be differences in the evaluation of the energy in ChemInt with respect to the SCF program. The energy difference between reconstructing the total energy from IQA components against the SCF energy is a measure of the error of our method. For the evaluated molecular systems, this energy error per atom is always below chemical accuracy (see Figure 1) with a modest choice of integration parameters. The boundary is determined with a precision of more than 5 × 10^–4^ Å normal to the surface using an ODE integrator that preserves a local relative error of the same magnitude. The grid has 600 radial points and 5780 points distributed on a symmetric spherical *t*-design grid. A maximum expansion order of *l* = 4 (*l* = 6) for regions near (far from) the nucleus is chosen. Increasing the precision of the surface determination does not yield a better integration. As well, the length of the expansion chosen here is enough to approximate bielectronic integrals. From our observations, the quality of the grid is the most determining factor once the rest of the parameters have been fixed like here.

Nevertheless, it is important to realize that periodic systems require a significantly larger number of orbitals than molecular systems. Computations with maximum resolution are not always feasible, and one must find a balance of precision and speed. Therefore, we are interested in the minimum required computation needed to achieve a reasonable accuracy level. For all the systems tested, we found that a surface represented with approximately 6000 points is enough to integrate the total volume in the crystallographic unit cell with a volume error Δ*V*^cell^ = *∑*_*A*_*V*^*A*^ – *V*_cell_ below 0.200 Å^3^ if we combine it with a radial grid of at least 200 points (Table 3). The symmetrical *t*-design quadrature offers a more robust integration, devoid of occasional (large) volume integration errors present with Lebedev–Laikov grids. In particular, for half-Heusler LiMgN, the cell volume error would be 2.027 Å^3^ using a 5810-point Lebedev grid. Instead, it is 0.010 Å^3^ with a 5780-point *t*-design grid. The distribution of points offered by the new grids is more homogeneous, thus providing a better overall representation of basin surfaces. Besides this, volume errors take place at the boundary of the basins so the integration of the charge can, and this is the case from our observations, still be within chemically reasonable accuracy error (<0.01).

**Table 3:** Reconstructing the Crystallographic Unit Cell Volume from QTAIM Basin Volumes: Volume Error, Δ*V*^cell^, and Cell Charge, *Q*^cell^

compound	structure type	Δ*V*^cell^ [Å^3^]	*Q*^cell^ [e]
LiCl	NaCl	0.104	–0.005
NaCl	NaCl	0.168	0.009
MgO	NaCl	0.039	0.002
CsCl	CsCl	0.004	–0.000
C	diamond	–0.004	0.007
BN	zincbende	0.020	0.000
C	graphite	0.017	0.001
BN	BN-b	0.009	0.001
Na	α-W (bcc)	–0.028	0.001
Al	Cu (fcc)	0.010	0.000
MgB_2_	AlB_2_	0.014	0.002
LiMgN	MgAgAs	0.010	0.006
N_2_	β-N_2_	–0.007	0.008
CO_2_	CO_2_	–0.073	0.000

### Molecular Crystals

Molecular crystals are a suitable example to examine the type of strong bonds that one finds in molecules before shifting to the highly diverse bonds present in crystalline materials. For that matter, we analyze β-N_2_, and CO_2_ crystals. A priori, N–N and C–O bonds are only expected to differ slightly from a gas phase molecule. Indeed, covalent intramolecular energies in the solid phase bear a striking resemblance to bonds of equal internuclear distance between atoms in gas phase, as shown in Table 4. Intermolecular interactions in the solid barely debilitate the covalent stabilization provided by the N_2_ triple bonds. As well, the classic interaction *E*_cl_^NN^ between two nearest nitrogen atoms is almost unaltered. In this regard, the triple bond exhibited by a N_2_ molecule is transferable to a triple bond in β-N_2_.

**Table 4:** Comparison of IQA Two-Center Terms for Covalent Bonds in Molecular and Solid Phases β-N_2_ and CO_2_^a^

system	*A*–*B*	*R*^*AB*^	δ^*AB*^	*E*_nn_^*AB*^	*E*_ne_^*AB*^	*E*_ne_^*BA*^	*E*_Coul_^*AB*^	*E*_cl_^*AB*^	*E*_x_^*AB*^
N_2_ (mol.)	N–N	1.108	3.041	23.399	–21.944	–21.944	20.716	0.227	–0.906
N_2_ (solid)	N–N^(1)^	1.108	3.000	23.399	–21.952	–21.953	20.726	0.220	–0.903
CO_2_ (mol.)	C–O	1.149	1.396	22.114	–25.443	–13.413	15.453	–1.289	–0.444
CO_2_ (solid)	C–O^(1)^	1.149	1.323	22.114	–25.646	–13.152	15.269	–1.415	–0.426
CO_2_ (mol.)	O–O	2.297	0.433	14.743	–16.981	–16.981	19.563	0.343	–0.054
CO_2_ (solid)	O–O^(2)^	2.297	0.407	14.743	–17.081	–17.081	19.793	0.373	–0.052

a Nearest-neighbor interactions are denoted with *A*–*B*^(1)^, second neighbors with *A*–*B*^(2)^, and so on. Energies in Ha; distances in Å.

On the other hand, while the C–O bond is similar in both environments, the strong polarization of this bond permits ionic interactions with neighbor molecules, with O–O being the most intense interaction. The C–O bond is mainly altered by its classic ionic component, which varies by 0.126 Ha in comparison to 0.018 Ha for the covalent energy *E*_x_^CO^. In both scenarios, a strong contribution from dipolar and quadrupolar components to the classic energy is found. The same effect is observed for intramolecular O–O interactions but is more attenuated.

### Diamond and Zincblende Structure

#### Convergence of Bielectronic Integrals

**Figure 2:**
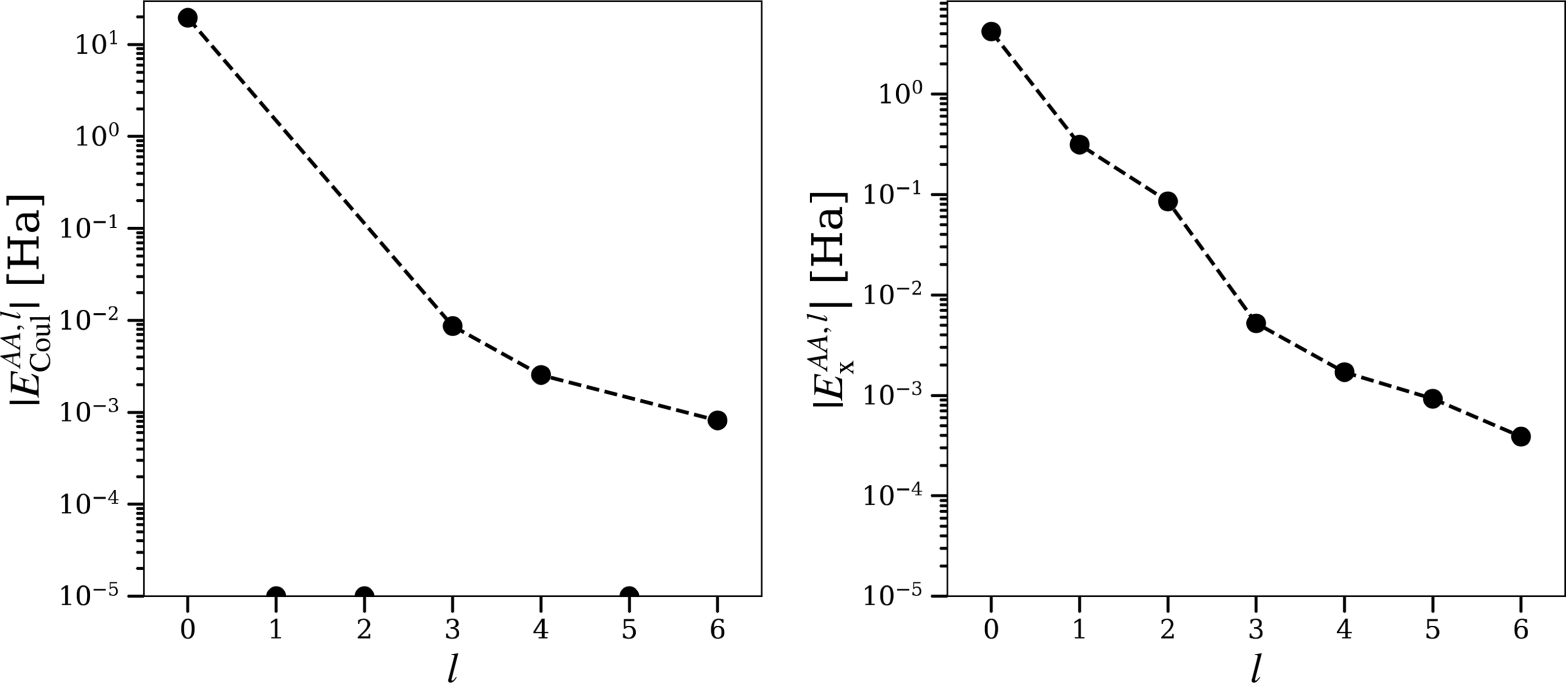
Diamond: Convergence of bielectronic integrals inside the basin of the carbon atom, with increasing multipolar order *l*,  and . Only their magnitude is plotted. Dashed lines indicate the trend of convergence for symmetry allowed terms. Disconnected dots are not allowed by symmetry and are nonzero due to numerical errors. Terms below 10^–5^ Ha are represented as dots at the bottom.

**Figure 3:**
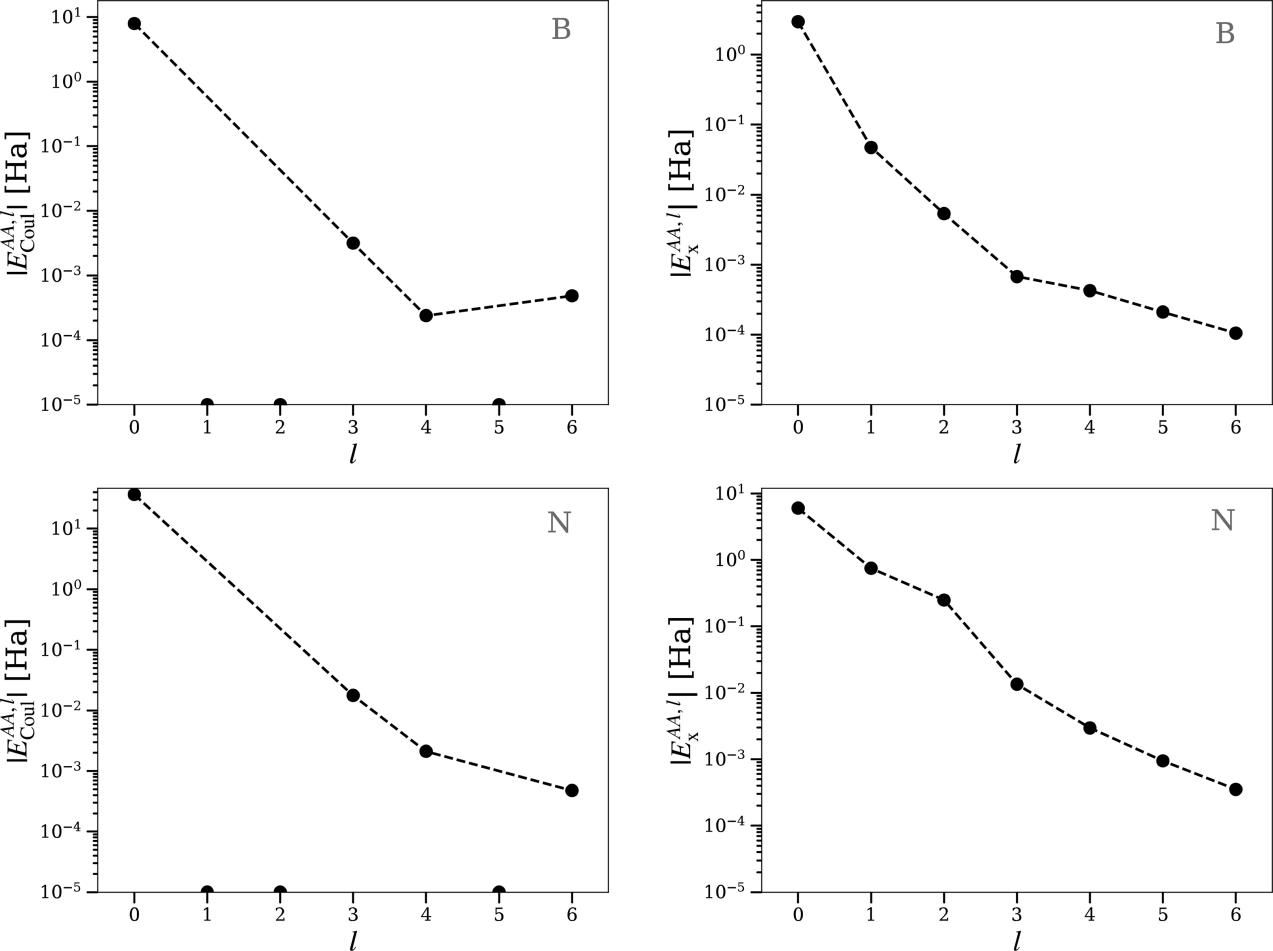
BN (zincblende): Convergence of bielectronic intrabasin integrals with increasing multipolar order *l*. For point and lines explanations, see Figure 2.

A steady convergence with increasing multipolar order is observed for intra-atomic bielectronic integrals in diamond and zincblende BN (Figures 2 and 3). The Coulomb energy *E*_Coul_^*AA*^ shows a dominating monopolar term (*l* = 0) followed by zero *l* = 1, 2 terms as demanded by symmetry.^48^ The *l* = 3, 4 terms are nonzero due to nonspherical distribution of electrons in the basin. The *l* = 5 term vanishes again due to symmetry. These observations for diamond atoms are in close agreement with those of a carbon atom in methane or the quaternary atom in neopentane (cf. Supporting Information, Figure S2 and Figure S3).

**Figure 4:**
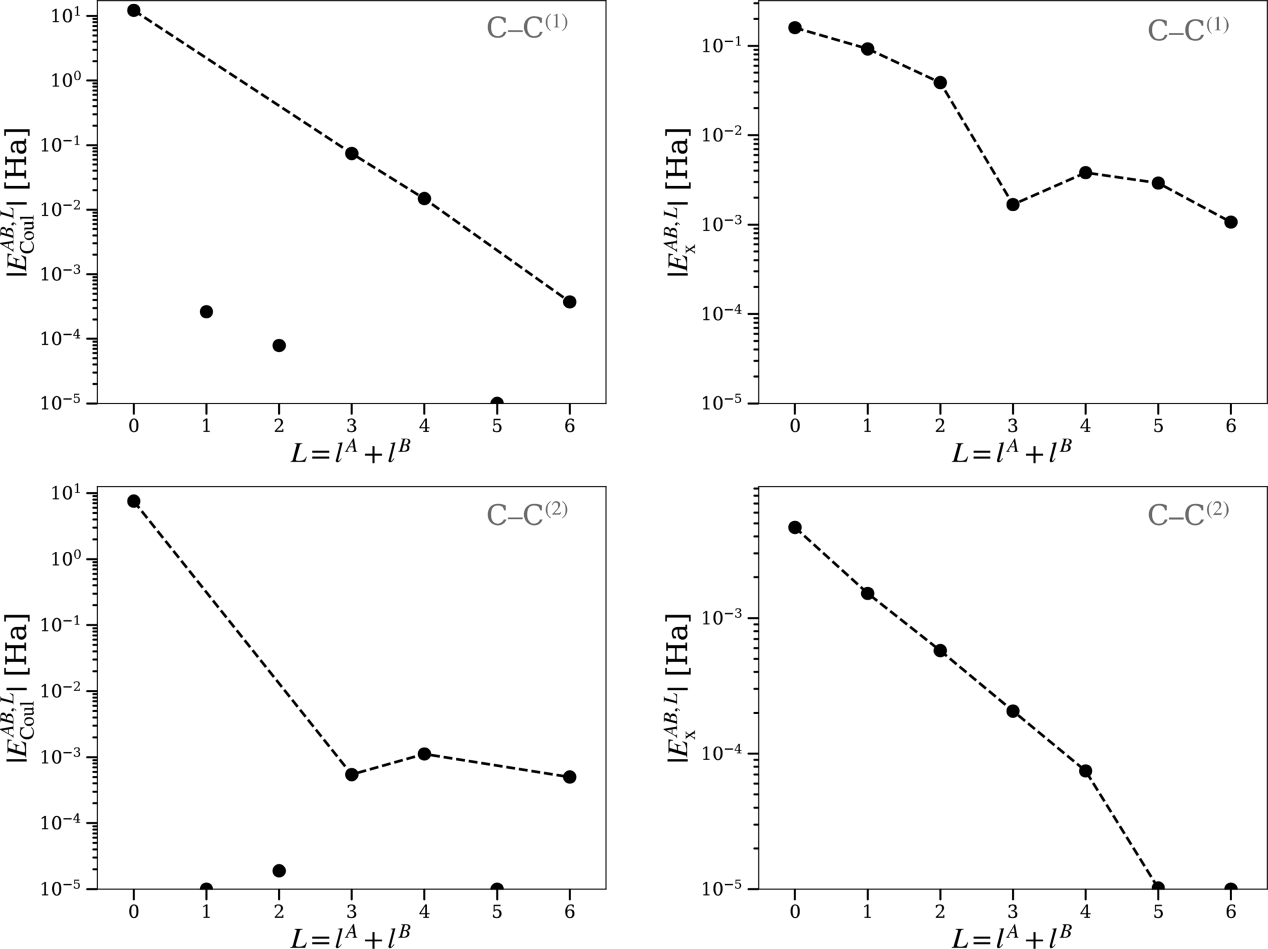
Diamond: Convergence of bielectronic interbasin integrals with increasing bipolar order *L* = *l*^*A*^ + *l*^*B*^. Labels in the top right indicate an interaction of atom *A* with an atom *B* of the *i*th coordination sphere as *A*–*B*^(*i*)^.

Convergence with *l* of nearest-neighbor inter-atomic electron–electron terms in diamond is slower than for intra-atomic terms (see Figures 2 and 4). The convergence of Coulomb and exchange components with the order of the multipolar expansion was examined to assess the possibility of truncating the series. Storage of higher-order terms contributes to a substantial increase of computation time, specially due to the increasing number of averaged overlap densities *R*_*l*,*m*_^*i*,*j*^. Coulomb and exchange terms show the expected convergent behavior. However, terms of order *l* = 3, 4 are still important for the Coulomb integral. This forces us to take several orders in the expansion. This behavior could, as well, vary for other systems. Therefore, it is only a preliminary observation.

**Figure 5:**
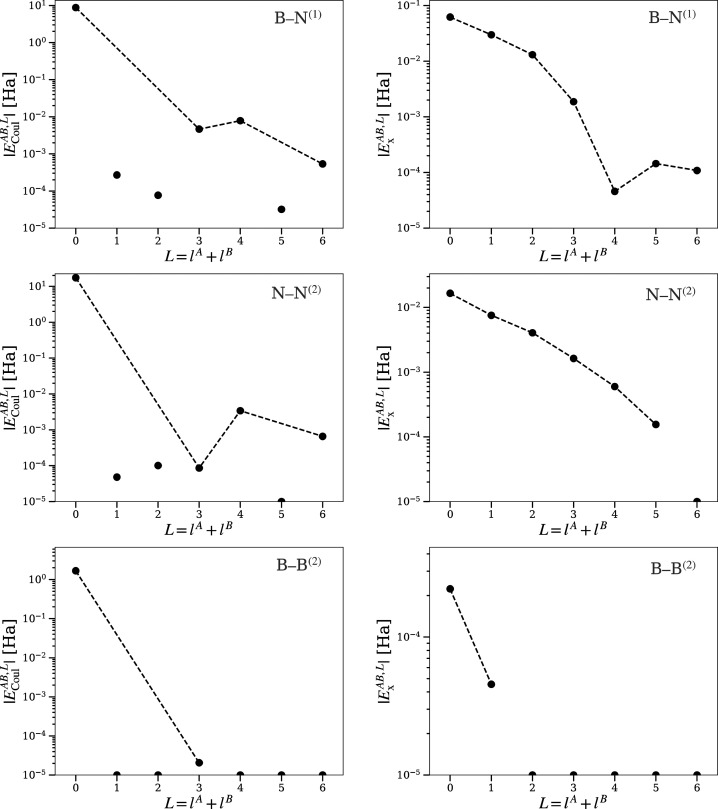
BN (zincblende): Convergence of bielectronic interbasin integrals with increasing bipolar order *L* = *l*^*A*^ + *l*^*B*^. For point and lines explanations, see Figure 2.

The Coulomb integral *E*_Coul_^*AB*^ between a boron atom and a nearest N atom is well approximated from the (*l*^*A*^, *l*^*B*^) = (0, 0) term (Figure 5). Higher order terms *L* = *l*^*A*^ + *l*^*B*^ > 0 clearly reflect the characteristics of the electron distribution in their respective basins. Terms with order *L* = 1, 2 result from the combinations {(0, 1), (1, 0), (1, 1), (2, 0), (0, 2)}. As we have seen above, since the symmetry in B and N sites is tetrahedral, *l* = 1, 2 terms are zero (Figure 3). The same argument justifies the absence of *L* = 5 terms. Thus, only with *L* = 0, 3, 4, and 6 are the corrresponding bicentric Coulomb terms nonzero. Only due to numerical accuracy are they nonzero. More distant interactions like the closest N–N and B–B exhibit the same pattern, but their contribution from higher *L* order terms is already small, especially for B–B interactions.

#### Self-Energies and Near Neighbor Interactions

Having a generic method to decompose atomic energies allows us to compare molecular and solid systems on the same footing. Diamond is a prototype of a covalent network with bonded quaternary carbon atoms. A carbon atom with the same coordination in a molecule is the quaternary carbon of neopentane.

Table 5 compares the population and intra-atomic energy components of this atom in the two environments. The average electron population of the quaternary C atom in neopentane is slightly lower than that in diamond carbon atoms. Both intra-atomic classic *E*_cl_^CC^ and exchange energies *E*_x_^CC^ are larger in diamond due to the higher population of the atomic basins.

**Table 5:** IQA Monocentric Integrals Inside Basins with *T*_*d*_ Point Symmetry^a^

system	*A*	⟨*N*_e_^*A*^⟩	λ^*A*^	σ^2^(*A*)	*T*^*A*^	*E*_ne_^*AA*^	*E*_Coul_^*AA*^	*E*_cl_^*AA*^	*E*_x_^*AA*^
neopentane	C	5.929	3.775	2.154	37.781	–89.694	19.154	–70.540	–4.566
diamond	C	5.999	3.820	2.180	37.917	–90.128	19.522	–70.606	–4.602
BN	B	2.839	2.075	0.765	23.700	–51.753	7.923	–43.830	–3.008
	N	9.161	7.517	1.643	55.650	–138.162	36.638	–101.524	–7.019

a Energies in Ha units.

Carbon atoms have a similar inter-atomic distance in diamond and neopentane, approximately 1.545 Å. To facilitate the discussion, the C – C distance in neopentane is fixed to be exactly the same as in diamond. This way, the nucleus–nucleus repulsion is identical. Full relaxation of the geometry does not change our conclusions. Inter-atomic interaction energies are presented in Table 6. As for intra-atomic terms *E*_Coul_^CC^, electron–electron Coulomb energies are larger in the solid phase. This is counterbalanced by *E*_ne_^CC^ to finally yield a smaller classic energy. In diamond, electron–nucleus interactions are symmetrical, *E*_ne_^*AB*^ = *E*_ne_^*BA*^. The sum of all classic electrostatic terms nearly cancels to yield *E*_cl_^CC^ = 0.019 Ha for neopentane and *E*_cl_^CC^ = 0.014 Ha for diamond because the electroneutrality of the atom makes the *L* = 0 term vanish. The classical *L* > 0 energy term *E*_cl,*L*≠0_^*AB*^ has to be positive for neutral nonoverlapping charge distributions.^8^

**Table 6:** IQA Two-Center Terms for Relevant Interactions in Neopentane, Diamond, and Zincblende BN^a^

system	*A*–*B*	*m*	*R*^*AB*^	δ^*AB*^	*E*_nn_^*AB*^	*E*_ne_^*AB*^	*E*_ne_^*BA*^	*E*_Coul_^*AB*^	*E*_cl_^*AB*^	*E*_x_^*AB*^
neopentane	C–C^(1)^	4	1.544	0.957	12.335	–12.058	–12.094	11.836	0.019	–0.288
diamond	C–C^(1)^	4	1.544	0.914	12.335	–12.217	–12.217	12.113	0.014	–0.284
	C–C^(2)^	12	2.522	0.042	7.554	–7.551	–7.551	7.549	0.001	–0.006
BN	B–N^(1)^	4	1.563	0.357	11.848	–15.502	–6.697	8.765	–1.586	–0.106
	N–N^(2)^	6	2.553	0.152	10.158	–13.290	–13.291	17.390	0.967	–0.030
	B–B^(2)^	6	2.553	0.002	5.183	–2.943	–2.943	1.671	0.968	–0.000

a is the number of equivalent interactions *A*–*B* per reference unit (*G* = C1C2 or *G* = B1N1) (eq 13). Energies in Ha; distances in Å units.

Exchange energies *E*_x_^CC^ are almost identical in neopentane and diamond. Conversely, the nearest-neighbor DI δ^CC^ in diamond is smaller due to higher delocalization of the wave function (more distant neighbors) in the solid.

Diamond presents a DI of δ^CC^ = 0.914 between nearest-neighbor atoms. The delocalization goes down to δ^CC^ = 0.044 for second nearest-neighbors. The variation of DIs for the third coordination shell obtained for equivalent interactions is too large to prevent a detailed numerical examination. Up to first and second nearest neighbors the sum rule (34) is shown in Table 7.

**Table 7:** Cumulative Electron Populations  of Atomic Basins in Diamond and Boron Nitride (Zincblende) from Successive Inclusion of Delocalization Shells α with DI δ_α_^a^

α	coordination sphere	δ_α_	⟨*N*_el_^*A*^⟩_cum_^α^
0	C	–	3.820
1	C–C^(1)^	0.914	5.648
2	C–C^(2)^	0.044	5.912
0	B	–	2.075
1	B–N^(1)^	0.357	2.789
2	B–B^(2)^	0.002	2.801
0	N	–	7.517
1	N–B^(1)^	0.357	8.231
2	N–N^(2)^	0.155	9.161

a The sum rule of eq 34 approaches ⟨*N*_el_^*A*^⟩=6 as delocalization with more distant atoms is included. Boron has ⟨*N*_el_^*A*^⟩ = 2.839 electrons, and nitrogen has ⟨*N*_el_^*A*^⟩ = 9.161 electrons.

Boron nitride represents an example of a diamond-type structure with polar bonds. While the nearest-neighbor electrostatic interaction *E*_cl_^CC^=0.014 Ha in diamond represents a destabilization, in B–N it is a stabilizing classic interaction between opposite charged atoms with an energy contribution of *E*_cl_^*BN*^=–1.586 Ha (Table 6).

The nearest-neighbor exchange interaction *E*_*x*_^*BN*^ is only one-third of the analogous nearest neighbors interaction *E*_*x*_^CC^ in diamond. Note that this decrease can be estimated (31) from the strong decrease of the delocalization index from δ^CC^ = 0.957 to δ^*BN*^ = 0.357.

For BN, in overall, classic electrostatic energies between nearest neighbors are much larger than the exchange ones. This fact reveals the high importance of classic interactions in the short-range regime.

The closest B–N interactions involve 0.36 electron pair sharing (Table 7) that parallels the value of 0.37 obtained before.^49^ Nitrogen atoms share 0.15 electron pairs with any other nearest nitrogen atom (to be compared with 0.35 electron pairs). The sum-rule for N is approached including interactions up to the second coordination shell (see Table 7). On the other hand, boron atoms have no significant electron sharing with other boron atoms. There are, however, 0.038 electrons still missing to recover the average number of electrons in the boron basin.

The localization indices are in agreement with results obtained before by some of us (Table 6).

#### Additive Interaction Energies

The additive interaction energy (cf. equation 11) for a 2-atom reference unit *G* = {C1, C2} ≡ C1C2 in diamond can be approximately obtained as follows. The covalent interaction energies  between nearest neighbors (−0.284 × 4 = −1.136 Ha) and  between second nearest neighbors (− 0.006 × 12 = −0.072 Ha) sum to a total covalent interaction energy of *E*_*x*_(C1C2) = −1.208 Ha. Higher neighbors are omitted due to a very small value of remaining bond fluctuations . A kind of lower bound of the neglected covalent interaction can be estimated with the zeroth order approximation if we assign all remaining electrons to the third coordination shell,  Ha. The stabilizing covalent interactions are counterbalanced by electrostatic interactions *E*_cl_(C1C2) = 0.014 × 4 + 0.001 × 12 = 0.068 Ha (in diamond) from contributions between first and second nearest neighbors, respectively. The total interactions considered amount to *E*_int_(C1C2) = −1.140 Ha.

For zincblende, again, a diatomic reference unit *G* = {B1, N1} ≡ B1N1 is chosen. The approximate covalent interaction energy up to second nearest neighbor terms sums nearest neighbor B–N^(1)^ interactions (− 0.106 × 4 = −0.424 Ha), second nearest neighbors N–N^(2)^ interactions (− 0.030 × 6 = −0.180 Ha), and second nearest neighbor B–B^(2)^ interactions (0.000 × 6 = 0 Ha) giving *E*_*x*_(B1N1) = −0.604 Ha. While the electron sum rule is already well satisfied for N, some 0.038 electrons are missing on B. If we use this number to approximate the maximum additional covalent energy, the third nearest neighbor B–N^(3)^ interaction with a distance  may be estimated to contribute maximally . It is interesting that a non-negligible covalent N–N^(2)^ interaction has been found, which makes up 30% of the covalent bond energy. It can be considered as the residual bonding of the incompletely occupied N^2.161–^ atoms, which is a necessary consequence of the polar bonding B–N^(1)^ leading to the small DI δ^*BN*^ = 0.357.

The procedure applied for the covalent part is not feasible for the electrostatic part, because of the long-range of the electrostatic interactions and the well-known resulting nonconvergence of the simple coordination series energy in real space. The electrostatic part of the interaction per reference unit can only be approximated by the point charge approximation using the Madelung constant of zincblende *f*_*M*,*R*_ = 1.63806. The validity of the PCA for this procedure can be estimated by determining the PCA scale factor *s*_cl_ for the computed first and second nearest neighbor *E*_cl_(B1N1) energies using *Q* atomic net charges and the corresponding distances , and . It turns out that the PCA energy values are rather close to the exact IQA ones (, ), such that the PCA is well justified. The Madelung energy per unit cell *G* = B1N1 is calculated according to  Ha, where *R* is the first-neighbor B–N distance. Although *l* = 1, 2 (dipoles, quadrupoles) are forbidden by symmetry, there are contributions from classic electrostatic terms with *L* > 0 due to the large atomic net charges present in the system. Owing to their absence in the Madelung formula, they must be accounted at least for nearest neighbors. We compute them with *E*_cl_(*G*) – *E*_cl,*L*=0_(*G*) = −0.026 Ha, where the last term is just the point-charge contribution (eq 30). The total electrostatic energy is then *E*_cl_ = −2.617 Ha. From these values, the total interaction energy amounts to *E*_int_(B1N1) = −3.221 Ha. The fraction of covalent bond energy per *G* = B1N1 with respect to the total interaction energy  yields a 19% contribution of covalent interaction. Based on this fraction, the interaction in BN (zincblende) is characterized by dominating 81% ionic interactions. Still, the covalency is non-negligible, and it represents the local type of bonding, while the electrostatic Coulomb interaction is a collective type of bonding as discussed previously.^50^ While the covalent bonding A–B is a local property of A and B, the electrostatic interaction A–B has long-range consequences, alternating stabilizing and destabilizing terms. These two different types of bonding cannot be discussed on the same footing, i.e., in a local picture. For this reason, the energies per reference unit have been calculated for characterization.

### Honeycomb Networks

Honeycomb networks can be found in graphite, hexagonal boron nitride, and the intermetallic superconductor MgB_2_. Table 8 shows nearest-neighbor interactions in the three hexagonal systems. In addition to the σ bonds discussed in the previous section, they might exhibit a certain degree of π double bond character. In graphite, it has been shown,^51^ based on delocalization indices, that the shortest C–C bonds with a DI δ^CC^ = 1.20 have partial double bond character, whereas in MgB_2_ the number of shared electron pairs is the same as for a single bond δ^*BB*^ = 1.00. For comparison, similar values of δ^CC^ = 1.202 in graphite and δ^BB^ = 0.983 in MgB_2_ are obtained.

**Table 8:** IQA Two-Center Terms for Relevant Interactions in Hexagonal Graphite, BN, and MgB_2_^a^

*A*–*B*	*m*	*R*^*AB*^	δ^*AB*^	*E*_nn_^*AB*^	*E*_ne_^*AB*^	*E*_ne_^*BA*^	*E*_Coul_^*AB*^	*E*_cl_^*AB*^	*E*_x_^*AB*^
C–C^(1)*ab*^	3	1.423	1.202	13.391	–13.098	–13.102	12.846	0.037	–0.372
C–C^(2)*ab*^	6	2.464	0.054	7.731	–7.692	–7.692	7.655	0.002	–0.007
C–C^(3)*ab*^	3	2.845	0.037	6.696	–6.679	–6.681	6.665	0.001	–0.004
C–C^(4)*c*^	1	3.356	0.019	5.677	–5.709	–5.709	5.746	0.004	–0.003
C–C^(5)*c*^	6 + 3	3.645	0.006	5.227	–5.245	–5.247	5.266	0.001	–0.001
B–N^(1)*ab*^	3	1.446	0.452	12.810	–16.751	–7.065	9.244	–1.762	–0.135
B–B^(2)*ab*^	3	2.504	0.004	5.283	–2.936	–2.935	1.632	1.043	–0.000
N–N^(2)*ab*^	3	2.504	0.212	10.354	–13.604	–13.605	17.877	1.021	–0.040
B–N^(3)*ab*^	3	2.892	0.008	6.405	–8.419	–3.564	4.685	–0.893	–0.001
B–N^(4)*c*^	2	3.329	0.006	5.563	–7.329	–3.110	4.101	–0.776	–0.001
N–N^(5)*c*^	3	3.630	0.024	7.144	–9.411	–9.410	12.403	0.726	–0.003
B–B^(1)*ab*^	3	1.781	0.983	7.429	–8.392	–8.392	9.522	0.166	–0.249
B–B^(3)*ab*^	6	3.085	0.052	4.289	–4.953	–4.953	5.722	0.106	–0.006
B–B^(5)*ab*^	3	3.562	0.017	3.714	–4.303	–4.303	4.987	0.095	–0.002
Mg–B^(2)*c*^	12	2.504	0.061	12.681	–14.830	–10.964	12.824	–0.290	–0.013
Mg–Mg^(3)*ab*^	3	3.085	0.003	24.704	–21.364	–21.364	18.475	0.450	–0.000

a The reference units corresponding to  are *G* = C1C2, *G* = B1N1, and *G* = MgB1B2. Energies in Ha; distances in Å units. Key: *ab*, intralayer interaction; *c*, interlayer interaction

Graphite nearest-neighbor C–C bonds display higher *E*_*x*_^*AB*^ energies (covalent bond energies) than in the other systems with honeycomb networks. The nearest-neighbor B–N covalent bond is particularly weak. In the honeycomb layer, the *E*_*x*_ proportion of the B–N covalent bond compared to the C–C covalent bond, , parallels the proportion seen in diamond and zincblende BN, . Conversely, classic electrostatic interactions are different for each system studied here. BN is largely stabilized from collective classical interactions between ions. This finding provides conclusive support for interpreting B–N as a very polar bond. However, destabilizing second-neighbor ionic interactions overcome the stabilization achieved by nearest neighbors. In the long-distance limit, those classic ionic interactions would tend to partially cancel.^50^ Graphite only has high order multipole contributions to the classic electrostatic energy. Thus, already for first neighbors, a low value of *E*_cl_ = 0.037 Ha is obtained. In MgB_2_, nearest B–B interactions display a considerable classic (interionic) destabilization that is compensated by Mg–B classic electrostatic stabilization.

As in zincblende BN (*E*_*x*_^NN^ = −0.030 Ha), among second-neighbor interactions only N–N still has a notable exchange contribution *E*_*x*_^NN^ = −0.040 Ha in the hexagonal phase.

### Phase Stability: Cubic versus Hexagonal Structures

Diamond and graphite are the main allotropes of carbon. The experimental difference in enthalpies is known to be quite small, Δ*H*^298 K^ = +1.895 kJ/mol,^52^ requiring an exquisite degree of accuracy that challenges DFT functionals. Albeit the relative stability problem is already solved,^53^ the internal forces leading to a greater stability of graphite are not completely known. The additional stability might result from the delocalized π bonding framework. However, it is not clear if the higher coordinated carbon atoms (via σ bonds) in diamond should be less stable. The origin of graphite stability could as well be attributed to a particular local property like a different hybridization. In the spirit of a recent publication that includes those contributions in an analytical model,^54^ we perform a numerical exploration to elucidate why nature chooses graphite over diamond.

Similar to the case of diamond discussed in the Diamond and Zincblende Structure section, the case of graphite involves a combination of stabilizing covalent bond contributions  – 0.004 × 3 = −1.170 Ha for ortho, meta, and para neighbors for the chosen C1C2 reference unit. The covalent part of the C–C interaction in the *c* direction amounts to  =  Ha to be added to yield final *E*_*x*_(C1C2) = −1.182 Ha. The destabilizing electrostatic interactions amount to  =  = +0.126 Ha (intralayer) and  Ha (interlayer) summing up to *E*_cl_(C1C2) = +0.139 Ha. A total interaction energy of *E*_int_(C1C2) = −1.043 Ha is obtained. Comparison of the total interaction energy to that of the diamond modification *E*_int_(C1C2) = −1.140 Ha (*E*_*x*_(C1C2) = −1.208 Ha, *E*_cl_(C1C2) = +0.068 Ha) reveals that the covalent interactions up to third nearest neighbors are energetically smaller, and the destabilizing electrostatic ones are larger. This yields an interaction energy preference for the diamond structure of Δ*E*_int_(C1C2) = 0.097 Ha.

Thus, the interaction energies do not explain the obtained total energy difference Δ*E*^FHI-aims^(C1C2) = 0.009 Ha between both modifications giving a preference to the graphite modification. This finding suggests that, at least for the current calculation, the preference of the graphite modification is caused by intra-atomic electron reorganization terms. Indeed, inclusion of the intra-atomic energies using Δ*E*(C1C2) = *E*(C1C2, diamond) – *E*(C1C2, graphite) = Δ*E*_self_(C1C2) + Δ*E*_int_(C1C2) resolves this seeming discrepancy and yields the energetical preference of the hexagonal phases (Table 9). In the framework of Valence Bond Theory, one would argue, the *sp*^3^ hybridization of carbon in diamond requires more energy than the *sp*^2^ hybridization in graphite. Just as in the solid phase, the 4-connected central carbon atom in neopentane and the 3-connected one in phenalene display a consistent self-energy difference of Δ*E*_self_(2 × C1) = 0.068 Ha, thereby demonstrating that the hybridization of the carbon atom is related to the stability of graphite over diamond. This indicates that the relative stability of carbon allotropes is not ruled by nonlocal contributions (π delocalization) but by intra-atomic electron redistribution energies often coined as “hybridization energies”.

**Table 9:** Relative Phase Stability of Cubic and Hexagonal Phases for C and BN Compounds^a^

phase	*E*_*x*_(*G*)	*E*_cl_(*G*)	Δ*E*_int_(*G*)	Δ*E*_self_(*G*)^b^	Δ*E*(*G*)	Δ*E*^FHI-aims^(*G*)
C (cubic)	–1.208	0.068	0	0.146	0.049	0.009
C (hex.)	–1.182	0.139	0.097	0	0	0
BN (cubic)	–0.604	–2.617	0	B: 0	0.012	0.005
				N: 0.120		
BN (hex.)	–0.539	–2.645	0.037	B: 0.071	0	0
				N: 0		

a Values for a reference unit: *G* = C1C2 and *G* = B1N1. Δ*E*(*G*) values are referred to the most stable phase. Energies in Ha units.

b Due to numerical difficulties integrating the total kinetic energy with mHa accuracy, atomic kinetic energies *T*^*A*^ were scaled to recover the total kinetic energy from FHI-aims.

The covalent interaction part per diatomic unit *G* = B1N1 is calculated according to  =  = −0.528 Ha for ortho (3 B–N^(1)^), meta (3 B–B^(2)^ + 3 N–N^(2)^), and para neighbors (3 B–N^(3)^) respectively. The B–N^(4)^ and N–N^(5)^ interactions in the *c* direction amount to  Ha to yield a total covalent bond energy *E*_*x*_(B1N1) = −0.539 Ha. Since the Madelung constant for this structure is not fixed by symmetry, it cannot be found tabulated in the literature. Therefore, the ionic electrostatic interaction energy  Ha was computed with the Environ program using the QTAIM net charges. This value is slightly larger than  Ha obtained for cubic phase. The low symmetry of hexagonal BN allows classic contributions not present in the cubic phase *E*_cl,*L*>0_(B1N1) = 0.123 Ha that in sum counteract the point-charge Madelung energy. Thus, classic energy components account for *E*_cl_(B1N1) = −2.645 Ha. The portion of covalent interactions  of 17% is smaller than 19% obtained for the cubic variant. The total interaction energy obtained amounts to *E*_int_(B1N1) = −3.184 Ha, which is of smaller size than *E*_int_(B1N1) = −3.221 Ha obtained for cubic BN by 37 mHa. In contrast, the total energy difference per BN unit computed with FHI-aims is Δ*E*^FHI-aims^(B1N1) = 0.005 Ha energy preference to hexagonal BN. So, the total energies obtained from FHI-aims yield a preference for the hexagonal variants for both cases: carbon, and BN. In contrast, for both cases, the summed interaction energies yield a preference of the cubic structures. This seeming discrepancy is resolved again taking the intra-atomic energies into account as well. According to this finding it is the intra-atomic energies and not the interaction ones that are responsible for the preferred stability of the hexagonal phases (Table 9). For BN, an interesting competition between B and N species is found. Intra-atomic energies indicate a preference of B species for the zincblende environment, while N species prefer the hexagonal environment. In the end the higher stabilization of the N species in the hexagonal environment dominates, and from Δ*E*(B1N1) = *E*(B1N1, cubic) – *E*(B1N1, hex.), the preference for the hexagonal phase is obtained.

### Rocksalt-Type Structures

Ionic crystals should present situations with extremely polar bonds. As a case in point we consider LiCl, NaCl, and MgO.

Table 10 shows short-range interactions in the rocksalt-type structure for various compounds. Even nearest-neighbor inter-atomic interactions have a small energy contribution from electron sharing. One can foresee from the delocalization indices that this is the case. Classical interactions take over the exchange component for those systems. As expected from their large QTAIM charges, the classic energy only converges when long-range interactions are also included.

**Table 10:** IQA Two-Center Terms for Relevant Interactions in LiCl, NaCl, and MgO^a^

*A*–*B*	*m*	*R*^*AB*^	δ^*AB*^	*E*_nn_^*AB*^	*E*_ne_^*AB*^	*E*_ne_^*BA*^	*E*_Coul_^*AB*^	*E*_cl_^*AB*^	*E*_x_^*AB*^
Li–Cl^(1)^	6	2.565	0.044	10.523	–11.076	–7.374	7.762	–0.165	–0.007
Li–Li^(2)^	6	3.627	0.000	1.313	–0.920	–0.920	0.645	0.118	–0.000
Cl–Cl^(2)^	6	3.627	0.084	42.164	–44.393	–44.393	46.741	0.118	–0.012
Na–Cl^(1)^	6	2.896	0.063	34.173	–35.919	–31.452	33.061	–0.138	–0.010
Na–Na^(2)^	6	4.095	0.000	15.635	–14.391	–14.391	13.246	0.099	–0.000
Cl–Cl^(2)^	6	4.095	0.044	37.344	–39.274	–39.272	41.301	0.100	–0.005
Mg–O^(1)^	6	2.106	0.125	24.116	–29.218	–20.674	25.050	–0.725	–0.029
Mg–Mg^(2)^	6	2.979	0.001	25.579	–21.931	–21.931	18.803	0.520	–0.000
O–O^(2)^	6	2.979	0.090	11.368	–13.803	–13.803	16.759	0.522	–0.014

a The net charges of QTAIM atoms are ±0.90*e*, ± 0.88*e*, and ±1.71*e* in LiCl, NaCl, and MgO, respectively. Energies in Ha; distances in Å units.

The additive energy for a reference unit *G* = LiCl is computed as follows. The covalent interaction energy from interactions with the first and second coordination sphere is *E*_*x*_(LiCl) = −0.007 × 6 + 0.0 × 6–0.012 × 6 = −0.114 Ha where six Li–Cl^(1)^, six Li–Li^(2)^, and six Cl–Cl^(2)^ interactions are considered. The lattice electrostatic energy assuming point charges is  Ha based on a Madelung summation. The effects of nonsphericity of the charge distribution around ions has an effect that is appreciated only for nearest neighbor interactions, as seen from their scaling factors of *s*_cl_^(1)^=0.98 and *s*_cl_^(2)^ = 1.00. To correct the classic energy for first-neighbors, Li–Cl^(1)^, higher order multipolar contributions, taken from the IQA inter-atomic energy as  Ha, are included. The total electrostatic energy is therefore *E*_cl_(LiCl) = −0.2848 Ha. Thus, the covalent part contributes  to the interaction energy *E*_int_(LiCl) = −0.398 Ha.

Similarly, for a *G* = NaCl reference unit, covalent interactions contribute with *E*_*x*_(NaCl) = −0.09 Ha. The classic energy, assuming point charges, is  Ha, that is corrected by nonspherical terms  Ha. The corrected classic energy *E*_cl_(NaCl) = −0.230 Ha entails a 72% percent of the interaction energy *E*_int_(NaCl) = −0.320 Ha.

MgO exhibits a stronger covalent stabilization than either LiCl or NaCl, *E*_*x*_(MgO) = −0.258 Ha. However, this increase is paralleled by stronger classical interactions  Ha generated by interacting point charges of ±1.71 *e*. The correction from nonspherical terms is also enhanced,  Ha. In overall, the total classic energy is *E*_cl_(MgO) = −1.215 Ha. Thus, the covalent part contributes less, , to the interaction energy *E*_int_(MgO) = −1.473 Ha than the previous systems with rocksalt-type structure.

With respect to interaction energies, the sequence of increasing covalent character is MgO (17%) < NaCl (28%) < LiCl (29%).

### Scaled Point-Charge Approximation (sPCA)

The kind of approximation discussed here has been used previously to predict the stability of phases with highly symmetric MgAgAs structure^6^ and to discuss Fe–Fe bonding in FeGa_3_.^4^

The validity of those approximations is tested against formally exact integrals from IQA method. Table 11 compares the zero order (point charge) approximation for interactions where largest deviations are expected, that is, between nearest neighbors.

**Table 11:** Scaled Point-Charge Approximation (sPCA) (See Eq 35) of Inter-Atomic Bielectronic Integrals for Nearest-Neighbor Interactions^a^

System	*R*^*AB*^	*Q*^*A*^	*Q*^*B*^	δ^*AB*^	*E*_cl_^*AB*^	*s*_cl_	*E*_x_^*AB*^	*s*_*x*_
β-N_2_	1.108	0.003	0.003	2.999	0.220	b	–0.903	0.630
CO_2_	1.149	2.280	–1.140	1.323	–1.415	1.182	–0.426	0.699
Graphite	1.422	0.000	–0.001	1.202	0.037	b	–0.372	0.832
BN (hex.)	1.446	2.214	–2.213	0.452	–1.762	0.982	–0.135	0.816
Diamond	1.545	0.001	0.001	0.914	0.014	b	–0.284	0.907
BN (cubic)	1.561	2.160	–2.160	0.357	–1.587	1.004	–0.106	0.881
MgB_2_	1.782	–0.813	–0.813	0.983	0.166	0.845	–0.249	0.852
MgO	2.106	1.711	–1.712	0.129	–0.725	0.984	–0.029	0.884
LiCl	2.566	0.897	–0.899	0.044	–0.165	0.991	–0.007	0.819
Al	2.863	0.000	0.000	0.273	0.0013	b	–0.054	1.071
NaCl	2.896	0.875	–0.878	0.064	–0.1376	0.980	–0.010	0.866
CsCl	3.540	0.826	–0.827	0.126	–0.1019	0.996	–0.017	0.915
Na	3.667	0.000	0.000	0.108	0.0008	b	–0.013	0.836

a Energies in Ha, distances in Å, and charges in *e* units.

b For interactions between (nearly) noncharged atomic species, the scaling parameters *s*_cl_ become quite large, because the electrostatic interaction is then no longer dominated by a monopolar  term. Nevertheless, the absolute error of this assumption is typically small, because the interactions are weak.

Molecular crystals like β-N_2_ or CO_2_ present rather short interactions that can not be modeled with the point-charge approximation. Rather like molecules, both solids show very strong covalent bonds as evidenced by their DIs and inter-atomic *E*_x_^*AB*^. This is in agreement with chemical wisdom. Covalent bonds break the spherical symmetry of the density leading to larger contributions from higher order multipoles to the classic energy. Even more, in N_2_ the atomic charge is zero but there is still a 0.220 Ha classic destabilization.

Graphite and diamond feature fractional double bonds and covalent single bonds, respectively, which display a corresponding increase of inter-atomic distances that leads to a corresponding decrease of *E*_cl_^CC^.

An inflection point is seen for zincblende BN. Despite having also covalent single bonds like diamond, the classic energy closely follows the point-charge approximation *s*_cl_ ≈ 1, indicating that the electron density is close to the spherical one.

Ionic and metallic crystals typically display longer inter-atomic distances and conceptual bond orders <1. Therefore, their covalent and ionic interactions are expected to be accurately approximated with the simple scaled point-charge equations applying an intermediate scaling factor *s*_*x*_ ≈ 0.75–0.85.

## Conclusions

The new software package ChemInt was developed to decompose the energy of crystalline solids according to the Interacting Quantum Atoms approach. The QTAIM basins are chosen as domains for the energy decomposition. Other possible spatial partitions (i.e., Becke fuzzy atoms, Hirshfeld atoms, ...) deserve future research and will assess the robustness of our conclusions. A number of key issues were addressed which limited the accuracy of the integration. The package ChemInt was used for the examination of some prototype crystalline solids. For the ionic solids studied, covalent interactions contribute 15–30% to the interaction energy. The scale parameters of our scaled point-charge approximation were obtained from the formally exact integration of covalent and classic inter-atomic interaction energies. While certain trends for these parameters are already visible, further investigations are needed to faithfully predict them for all kinds of bonding situations. The phase stability of graphite over diamond and hexagonal BN over zincblende BN was obtained in terms of chemically meaningful energy components. Future explorations along this line are a promising area of research. The obvious limitations of the application to very large systems can be significantly reduced in the future with the exploitation of crystal symmetries and treating only valence electrons. The new program may contribute toward a better understanding of interactions in crystalline materials, in particular in those which do not follow traditional valence rules. Extending the IQA method to planewave bases is perfectly possible, and it can be implemented on any electron structure package because it does not depend on the representation of the wave function in terms of atomic orbitals.

## Supporting Information Available

Computational details of ChemInt calculations and supporting IQA results (PDF)
