# Supplementary material for: Interacting Quantum Atoms Method for Crystalline Solids
Source: J Phys Chem A. 2021 Oct 1;125(40):9011–25. doi: 10.1021/acs.jpca.1c06574 (PMC8521528; doi:10.1021/acs.jpca.1c06574)
Supplement: Supplementary file 1 — jp1c06574_si_001.pdf [file jp1c06574_si_001.pdf]

Supporting Information for "Interacting Quantum Atoms Method for Crystalline Solids"

Daniel Menéndez Crespo,*,† Frank Richard Wagner,*,† Evelio Francisco,‡ Ángel Martín Pendás,‡ Yuri Grin,† and Miroslav Kohout*,†

†*Max-Planck-Institut für Chemische Physik fester Stoffe, 01187 Dresden, Germany*

‡*Departamento de Química Física y Analítica, University of Oviedo, 33006 Oviedo, Spain*

E-mail: Daniel.MenendezCrespo@cpfs.mpg.de; Frank.Wagner@cpfs.mpg.de; Miroslav.Kohout@cpfs.mpg.de

ChemInt settings

ChemInt/Promolden parameters to integrate IQA energies for molecular systems:

- ODE solver: Bogacki-Shampine (order 3)
- ODE reltol = 10^{-3}
- ODE abstol = 10^{-5}
- Rmaxsurf = 5.0
- Quadratures: Gauss-Legendre + t-design
- Nrad = 300 in each basin region
- Nang = 5780 (outside β-sphere); 434 (inside)
- lmax = 6 (outside); 4 (inside)

ChemInt parameters to integrate IQA energies for solid state systems:

- ODE solver: Bogacki-Shampine (order 3)
- ODE reltol = 10^{-3}
- ODE abstol = 10^{-5}
- Rmaxsurf = 5.0
- Quadratures: Gauss-Legendre + t-design
- Nrad = 100 in each basin region
- Nang = 5780 (outside β-sphere); 434 (inside)
- lmax = 6 (outside); 4 (inside)

Additional IQA results

Diamond and hydrocarbon chains

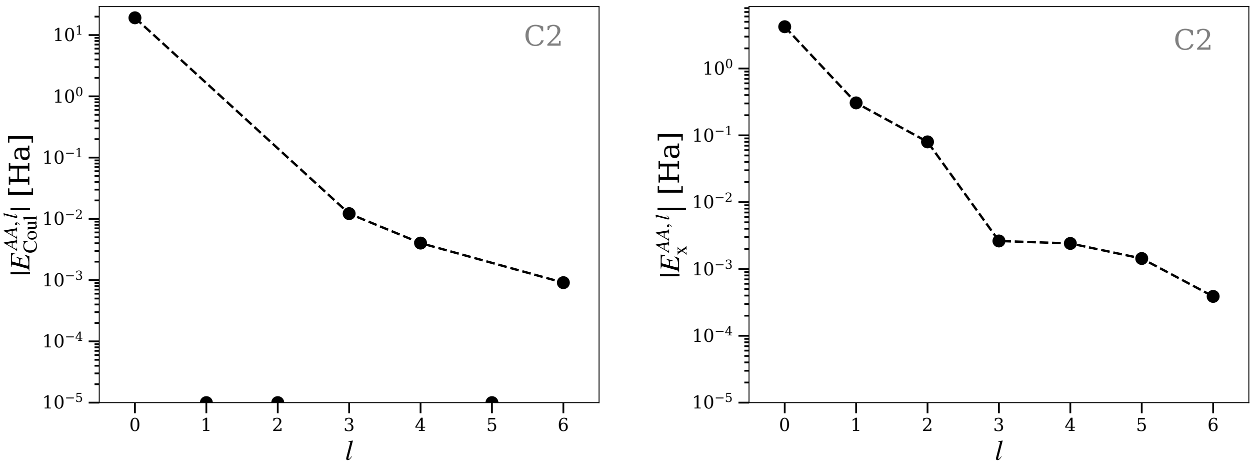

Figure S1: Methane: Convergence of bielectronic intra-basin integrals inside the carbon basin with increasing multipolar order l, $E_{\mathrm{Coul}}^{AA} = \sum_l E_{\mathrm{Coul}}^{AA,l}$ and $E_{\mathrm{xc}}^{AA} = \sum_l E_{\mathrm{xc}}^{AA,l}$. Points and lines explanations are given in the article.

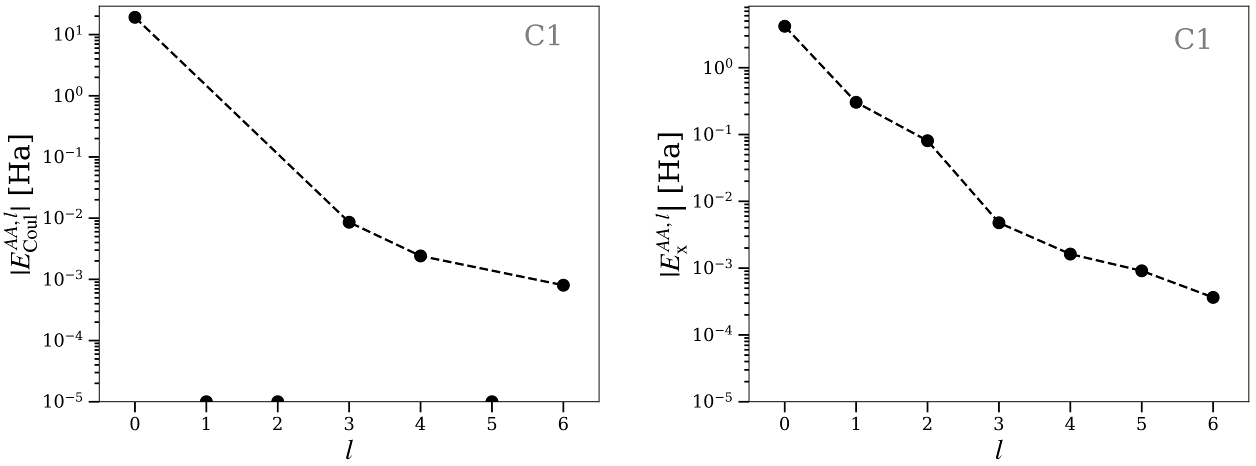

Figure S2: Neopentane: Convergence of bielectronic intra-basin integrals inside the carbon basin, $E_{\mathrm{Coul}}^{AA} = \sum_l E_{\mathrm{Coul}}^{AA,l}$ with increasing multipolar order l, $E_{\mathrm{xc}}^{AA} = \sum_l E_{\mathrm{xc}}^{AA,l}$.

Table S1: IQA monocentric terms inside a carbon basin in diamond, n-pentane (C1), n-butane (C1), propane (C1), ethane (C1), and methane (C1) (figures S4, S5, S6, S7, S8). Functional PBE, basis set: H(1s 2s 2p), C(1s 2s 2p 2p 3d 2s). Energy in Hartree units.

System	$\langle N_e^A \rangle$	λ^A	T^A [Ha]	E_{ne}^{AA} [Ha]	E_{Coul}^{AA} [Ha]	E_{xc}^{AA} [Ha]
Diamond	5.999	3.820	37.917	-90.128	19.522	-4.602
Neopentane	5.931	3.779	37.777	-89.691	19.156	-4.566
n-pentane	5.925	3.846	37.848	-89.799	19.162	-4.584
n-butane	5.918	3.843	37.833	-89.756	19.129	-4.581
n-propane	5.911	3.839	37.817	-89.709	19.092	-4.577
Ethane	5.930	3.907	37.816	-89.736	19.100	-4.590
Methane	5.962	3.993	37.816	-89.796	19.138	-4.607

Table S2: IQA C−C bond energetics in diamond and hydrocarbon molecules. Distances in Å units.

System	R^{AB} [Å]	δ^{AB}	E_{nn}^{AB} [Ha]	E_{ne}^{AB} [Ha]	E_{ne}^{BA} [Ha]	E_{Coul}^{AB} [Ha]	E_{xc}^{AB} [Ha]	A–B^a
Diamond	1.545	0.914	12.335	-12.217	-12.217	12.113	-0.284	C–C′
neopentane	1.545	0.956	12.312	-12.042	-12.070	11.819	-0.288	C1–C2
n-butane	1.524	0.992	12.493	-12.200	-12.164	11.894	-0.308	C2–C5
n-propane	1.524	0.992	12.492	-12.197	-12.149	11.878	-0.308	C2–C5
Ethane	1.524	1.010	12.495	-12.174	-12.174	11.879	-0.310	C2–C5

[a] Atom labels in the Supporting Information, figures S3, S5, S6, S7.

Table S3: High order multipolar contributions to the classic energy of BN phases is shown in detail here. Species have a $Q = \pm 2.161$ charge in the cubic phase and $Q = \pm 2.214$ in the hexagonal phase. The reference unit is $G =$ B1N1. $E_{\mathbf{cl},L>0}^{AB}(G) = m^{(AB)^i}\left(E_{\mathbf{cl}}^{AB} - \frac{Q^A Q^B}{R^{AB}}\right)$. Cubic BN is stabilized by -25 mHa and hexagonal BN is destabilized by 124 mHa.

Phase	A–B	m	R^{AB} [Å]	E_{cl}^{AB}	$E_{cl,L>0}^{AB}(G)$
Cubic	B–N$^{(1)}$	4	1.563	-1.586	-0.0197
	B–B$^{(2)}$	6	2.553	0.968	0.0002
	N–N$^{(2)}$	6	2.553	0.967	-0.0058
Hex.	B–N$^{(1)}$	3	1.446	-1.762	0.0956
	B–B$^{(2)}$	3	2.504	1.043	0.0212
	N–N$^{(2)}$	3	2.504	1.021	-0.0447
	B–N$^{(3)}$	3	2.892	-0.893	0.0117
	B–N$^{(4)\,c}$	2	3.329	-0.776	0.0063
	N–N$^{(5)\,c}$	3	3.630	0.726	0.0342

Table S4: Total energy components of BH_3 computed with ChemInt, Promolden, and GAMESS. The evaluated number of electrons per formula unit are 8.0001 (ChemInt), 7.9998 (Promolden). E_x was re-scaled from the integration of the PBE functional.Energy in Ha units.

Energy	ChemInt	Promolden	GAMESS
Total energy, E	-26.552	-26.552	-26.552
Kinetic energy, T	26.355	26.355	26.355
Total potential energy, E_{ne}	-75.511	-75.509	-75.510
Electron-electron energy, E_{ee}	15.148	15.145	15.146
Coulomb energy, E_{Coul}	20.216	20.213	20.214
Exchange-correlation energy, E_{xc}	-5.068	-5.068	-5.068

Table S5: Total energy components of methane computed with ChemInt, Promolden, and GAMESS. The evaluated number of electrons per formula unit are 10.0001 (ChemInt), 9.9998 (Promolden). E_x was re-scaled from the integration of the PBE functional.Energy in Ha units.

Energy	ChemInt	Promolden	GAMESS
Total energy, E	-40.462	-40.461	-40.463
Kinetic energy, T	40.216	40.216	40.216
Total potential energy, E_{ne}	-120.252	-120.250	-120.252
Electron-electron energy, E_{ee}	26.040	26.040	26.039
Coulomb energy, E_{Coul}	32.881	32.880	32.880
Exchange-correlation energy, E_{xc}	-6.840	-6.840	-6.840

Atomic basins and labels

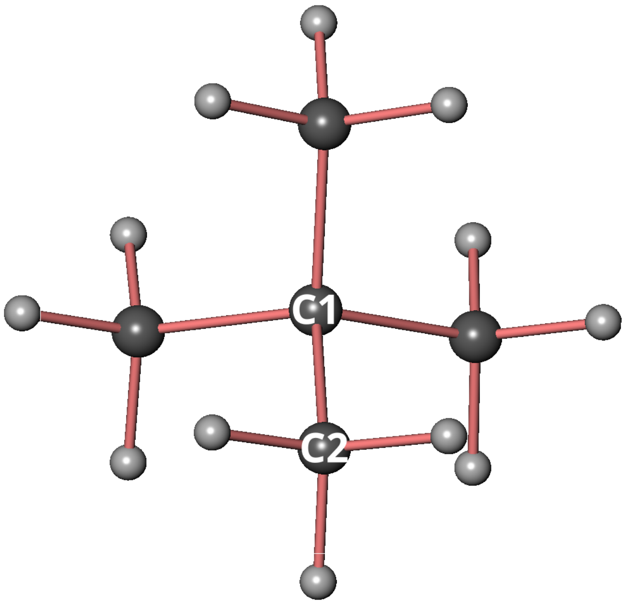

Figure S3: Labeling of atoms in neopentane.

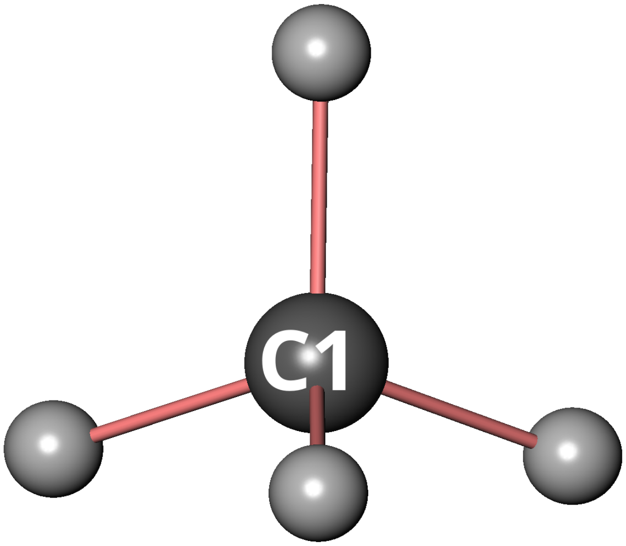

Figure S4: Labeling of methane atoms.

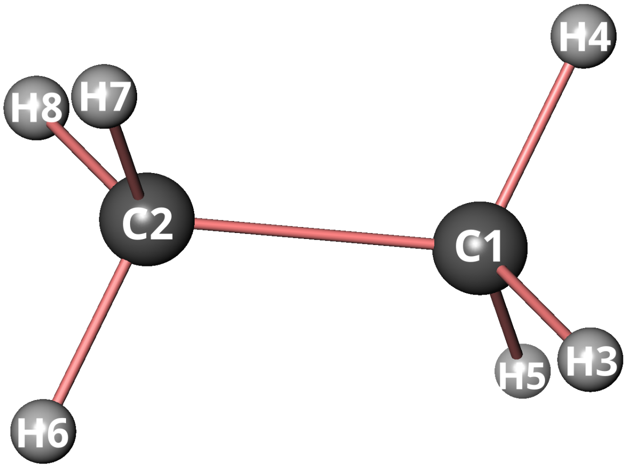

Figure S5: Labeling of ethane atoms.

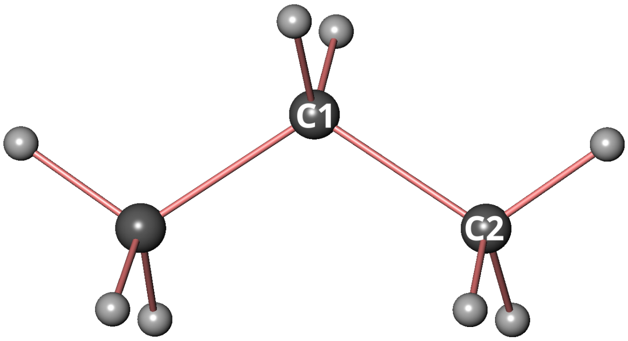

Figure S6: Labeling of n-propane atoms.

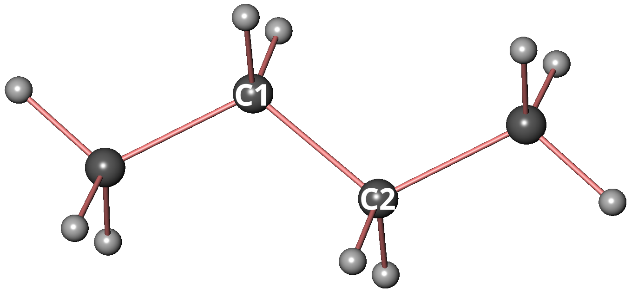

Figure S7: Labeling of n-butane atoms.

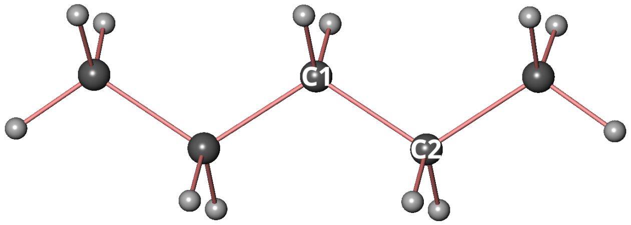

Figure S8: Labeling of n-pentane atoms.
